# Digital Tools in Behavior Change Support Education in Health and Other Students: A Systematic Review

**DOI:** 10.3390/healthcare10010001

**Published:** 2021-12-21

**Authors:** Lucija Gosak, Gregor Štiglic, Leona Cilar Budler, Isa Brito Félix, Katja Braam, Nino Fijačko, Mara Pereira Guerreiro, Mateja Lorber

**Affiliations:** 1 Faculty of Health Sciences, University of Maribor, 2000 Maribor, Slovenia; lucija.gosak2@um.si (L.G.); gregor.stiglic@um.si (G.Š.); leona.cilar1@um.si (L.C.B.); nino.fijacko@um.si (N.F.); 2 Faculty of Electrical Engineering and Computer Science, University of Maribor, 2000 Maribor, Slovenia; 3 Usher Institute, University of Edinburgh, Edinburgh EH8 9YL, UK; 4 Nursing Research, Innovation and Development Centre of Lisbon, Nursing School of Lisbon, 1600-190 Lisbon, Portugal; isafelix@esel.pt (I.B.F.); mara.guerreiro@esel.pt (M.P.G.); 5 Faculty of Healthcare, Sports and Welfare, Inholland University of Applied Sciences, 3521 Haarlem, The Netherlands; katja.braam@inholland.nl

**Keywords:** digital tools, didactics, noncommunicable diseases, chronic diseases, behavior change support education, health science

## Abstract

Due to the increased prevalence of chronic diseases, behavior changes are integral to self-management. Healthcare and other professionals are expected to support these behavior changes, and therefore, undergraduate students should receive up-to-date and evidence-based training in this respect. Our work aims to review the outcomes of digital tools in behavior change support education. A secondary aim was to examine existing instruments to assess the effectiveness of these tools. A PIO (population/problem, intervention, outcome) research question led our literature search. The population was limited to students in nursing, sports sciences, and pharmacy; the interventions were limited to digital teaching tools; and the outcomes consisted of knowledge, motivation, and competencies. A systematic literature review was performed in the PubMed, CINAHL, MEDLINE, Web of Science, SAGE, Scopus, and Cochrane Library databases and by backward citation searching. We used PRISMA guidelines 2020 to depict the search process for relevant literature. Two authors evaluated included studies using the Mixed Methods Appraisal Tool (MMAT) independently. Using inclusion and exclusion criteria, we included 15 studies in the final analysis: six quantitative descriptive studies, two randomized studies, six mixed methods studies, and one qualitative study. According to the MMAT, all studies were suitable for further analysis in terms of quality. The studies resorted to various digital tools to improve students’ knowledge of behavior change techniques in individuals with chronic disease, leading to greater self-confidence, better cooperation, and practical experience and skills. The most common limitations that have been perceived for using these tools are time and space constraints.

## 1. Introduction

Due to the growing burden of chronic diseases, such as obesity, diabetes, and cardiovascular disease [1,2], the need for individual support in self-management is increasing [3,4,5]. Changing behaviors for effective self-management can improve health outcomes and the quality of life of people with chronic diseases [1]. Furthermore, it can improve life expectancy and reduce health costs [6]. New best practice and evidence-based healthcare education are needed to prepare future healthcare and other professionals to support people with chronic disease, which reflects a significant challenge for educators [7].

Healthcare and other professionals have a key role in promoting healthy behavior and motivating individuals with chronic diseases to live healthier lives [8]. They can signal problems, provide tailored information, enable persons with chronic disease to participate in lifestyle supporting programs, and help these persons maintain healthy behaviors [9]. Public health has advanced in recent years in solving complex systemic problems. Healthcare and other professionals must be equipped with a range of skills such as reducing complications, problem-solving and evidence-based practice, and decision-making [10,11].

The quality of education provided to patients with chronic disease is significantly influenced by the quality of academic education students receive, i.e., education about professional roles, supervision received, self-preparation for training, mutual peer support, and teaching instruments [12]. Therefore, learning by health and other students is extremely important. Clinical learning is one of the essential issues that help understand students’ practice in the clinical health environment and influences their professional development [13]. Nieman (2007), for example, believed that educational modules in chronic diseases should offer appropriate training for students [14]. Students who also have an education in behavioral or social sciences will find it easier to identify risky behaviors of persons with chronic disease and appropriately encourage behavior changes [15].

Despite the importance of health education, the literature suggests the existence of insufficient competencies of healthcare and other professionals in this field [16,17,18]. As part of the Train4Health project (https://www.train4health.eu/, accessed on 1 November 2021), we want to improve students’ education in supporting behavior changes to promote self-care effectively in people with chronic diseases. The project target groups are nursing, pharmacy, and sport sciences students.

There is a need for digital teaching tools, such as simulation software, e-learning, and digital guided courses, such as massive open online courses (MOOCs), to provide health education and behavior change support [17,18]. E-learning is defined as an educational intervention that is transmitted electronically over the Internet and requires various technological and communication systems. Among healthcare professionals, its use has increased significantly in recent years [19,20]. The Massive Online Open Course (MOOC) is an approach that uses the Internet to enable courses to achieve a broader educational impact to a wider range of individuals [21,22,23]. The use of technology in healthcare education makes it easier for students to acquire basic knowledge, skills, and psychomotor skills to improve decision-making competencies and practice in various events [24]. Using a combination of traditional teaching methods with e-learning methods can be an effective complement to improving their clinical skills [25].

The main objectives of this systematic review are to assess the outcomes of the use of digital teaching tools and review the assessment instruments to evaluate the research outcomes (e.g., education skills and learning experience) in health and other students following the introduction of digital teaching tools.

## 2. Materials and Methods

This systematic review was registered with PROSPERO (CRD42021233690). The review was performed following five steps: (1) formulating a review question, (2) identifying relevant work, (3) evaluating the quality of the study, (4) summarizing the evidence, and (5) interpreting the findings [26].

The first research question based on the PIO approach [27] was: “What is the outcome (O) of the use of digital teaching tools to support behavioral change (I) in healthcare and other professionals (P)?” Another research question was: “What assessment instrument are used (I) to assess research outcomes (e.g., education skills, learning experience, etc.) (O) in healthcare and other students?”.

The selection process for the relevant studies consisted of five steps: (1) databases search and backward citation search, (2) removal of duplicates, (3) screening of records based on the title and abstract, (4) overview of the results based on full text, and (5) analysis of studies involved in the synthesis.

A systematic search of the relevant literature took place in seven international databases: PubMed, CINAHL, MEDLINE, Web of Science, SAGE, Scopus, and Cochrane Library and with backward citation by manually searching the reference lists of all the articles included [28]. If individual records were not fully available or additional information about the results was required, we contacted the authors of the articles. We also searched for unpublished works in the application databases of various review protocols (PROSPERO).

The search in databases was performed using the following search string: (“nurs* student*” OR “healthcare student*” OR “pharmacy student*” OR “sport student*”) AND (“pedagogical method” OR “e-learning cours*” OR “online cours*” OR “MOOC” OR “case stud*” OR “simulation*” OR “virtual patient*”) AND (“knowledge*” OR “motivation*” OR “engagement*” OR “skill*” OR “competence*” OR “self-care” OR “self-management” OR “change the behavior” OR “change attitudes” OR “behaviour change” OR “behavior change” OR “behaviour change techniques” OR “behavior change techniques” OR “health behavior” OR “health behavior”) AND (“non-communicable disease” OR “chronic disease*” OR “chronic illness” OR “coronary disease” OR “coronary artery disease” OR “heart disease” OR “heart failure” OR “cardiovascular disease” OR “high blood pressure” OR “hypertension” OR “diabetes mellitus type 2” OR “ischemic heart disease” OR “type 2 diabetes” OR “non-insulin-dependent diabetes” OR “adult-onset diabetes” OR “NIDDM” OR “T2D” OR “obesity”). Search strategies for the individual databases are presented in Table S1. Database searches are presented in Table S2.

Based on the inclusion and exclusion criteria (Table 1), two authors screened the records individually. Articles reviewed in full text and excluded based on exclusion criteria are present in Table S3.

Two authors extracted the data from the relevant studies into a preprepared table with recoverable identification data (Table S4). To assess the quality of the studies, we used the Mixed Methods Appraisal Tool (MMAT) [29,30]. The MMAT is intended to critically evaluate studies included in systematic mixed methods reviews (qualitative, quantitative, and mixed studies) and enable methodological quality assessments. Mixed methods studying also includes individual evaluations of qualitative and quantitative methods. The quality assessment of the mixed method study should not exceed the quality of the weakest component. We reported our MMAT results in metrics and not as no metrics, as is described in the MMAT instructions, because of that way being more informative for the readers (Table S5) [29,30].

The findings were synthesized using a thematic method. The obtained results were classified into codes, subtopics, and main topics [31]. We also performed a content analysis of the relevant records [32].

## 3. Results

### 3.1. Results of Literature Review

**Figure 1:**
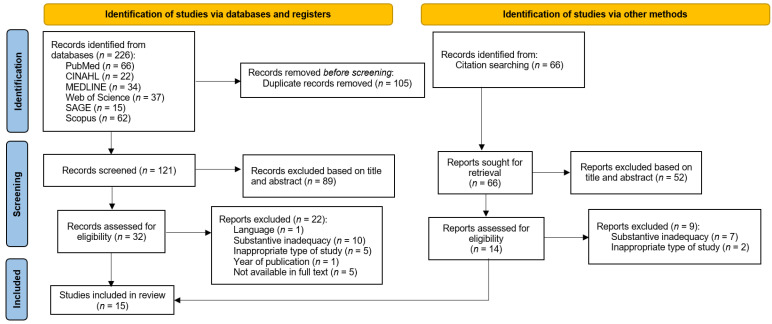
Flow diagram [34].

The search process for relevant results is shown in Figure 1 with a PRISMA flow diagram [33,34]. Fifteen studies were included in the final analysis based on the inclusion and exclusion criteria.

Table 2 presents the characteristics of included studies, study type, and MMAT score. All studies that received MMAT score of 50% or higher were included in the further analysis. Breakdown of MMAT Score is presented in Table S6.

The final analysis included six quantitative descriptive studies, one quantitative single-blind randomized controlled trial, one quantitative descriptive single-blind study, one-center, a cluster randomized controlled trials, one qualitative study, and six mixed study methods. The evaluation of studies using the MMAT ranged from 50% to 90%.

Participants learned to promote different techniques (e.g., MOOC, patient simulation, standard patients, etc.) of changing behaviors and treatment in the field of various disease states: diabetes (*n* = 3) [35,41,47], heart failure (*n* = 4) [37,43,49], COPD (*n* = 3) [38,40,45], stroke [38], asthma [49], prostate cancer [44], breast cancer [38], hypertension [43,46], mental health [48], and dementia [38] (each *n* = 1). They also address interventions related to behavior changes such as the transition of care [43], cardiac life support, insulin injection technique [39], use of inhalers [36], and care in an ambulance [42] (each *n* = 1). 

In the analyzed studies, the authors used a simulation (*n* = 12), a virtual case study (*n* = 1), and MOOC (*n* = 2) for teaching students and healthcare and other professionals with digital teaching tools. 

### 3.2. Assessment Instruments to Evaluate Research Outcomes

Table 3 includes information on the tools used to assess the outcomes in postgraduate students using a variety of digital behavioral change instruments (the basic data of the included studies and the main findings are presented in Table S4). All 15 studies used different instruments for evaluating the outcomes of the research. All the instrument descriptions are provided in Table 3.

### 3.3. Assessment of the Digital Teaching Tools Outcomes

The thematic analysis of the articles is presented below (Table 4).

The main themes we designed based on the thematic analysis are positive outcomes of using digital teaching tools and barriers to using digital teaching tools. Positive outcomes of using digital teaching tools include four subtopics: knowledge, confidence, practical experience, and collaboration. The findings in the articles showed that the use of digital technologies influences the active learning of users [38], developing skills [36,40], and critical thinking [38]. This also significantly impacts increasing their knowledge and maintaining their knowledge [39,49]. In this way, this also influences the improvement and building of self-confidence and self-confidence [38,48,49], as well as the increase in skills [41]. Thus, students are also more prepared for clinical and professional practices [35,44] and improve their professional network and cooperation with other professionals [35,37]. However, there are many restrictions on the use of digital tools, such as time constraints [43], financial barriers [43], and resource constraints such as space constraints [43] or material constraints [37]. In this study, the authors recommended providing more time for activity development.

**Table 4:** Thematic analysis.

Main Themes	Subthemes	Codes
Positive outcomes of using digital teaching tools	Knowledge	-knowledge retention [39,49] -increase in knowledge [39] -active learning [38] -developing/improving skills [36,40] -critical thinking [38] -significantly higher counseling [39]
	Confidence	-builds confidence [38] -felt more confident [48,49] -skills increased [41] -diabetes education skills assessed [41] -trust [45]
	Practical experience	-more prepared for interprofessional education [37] -improve the professional practice [35] -effect on their clinical/professional practice [44] -expressed satisfaction with experiencing such a practice [36]
	Collaboration	-increase their professional network [35] -think more positively about other professionals [37]
Barriers to the use of digital teaching tools	Restrictions	-using only one patient simulator [37] -time in students’ schedules [43] -financial resources [43] -space [43] -lagging feedback [46] -technology issues [46]
	Suggestions for improvement	-faculty time to develop activities [46]

## 4. Discussion

We included 15 articles in the final analysis. Of these, six were quantitative descriptive studies [35,37,41,45,47,49], two were randomized studies [36,39], six were mixed methods studies [40,42,43,44,46,48], and one was a qualitative study [38]. Different populations were included in the studies, such as nursing, sports science, and pharmacy students.

Simulations are among the most common digital teaching tools. Simulations in the undergraduate nursing curriculum are becoming increasingly popular and becoming the foundation of many nursing programs [57]. MOOC allows lecturers to reach a large, diverse audience. In a study using MOOC for the purpose of learning about health safety science, users reported a significant increase in competency. However, they pointed out that MOOC is difficult to include in all curricula [58].

Researchers are also advising the inclusion of digital badges and gamification digital teaching tools [59].

### 4.1. Assessment Tools

Based on the analyzed studies, we found that there is no unique tool that would allow insight and monitoring the effectiveness of different pedagogical approaches in students on their knowledge and effectiveness in supporting the changing behavior of a person with chronic disease. In individual studies, researchers used individual tools that monitor only individual aspects or are helpful only for individual diseases. Thus, for example, DAS-3 [53] is intended to assess self-confidence in education in developing skills in diabetes. Self-confidence can also be measured with the SSSC questionnaire [50,51]. Additionally, individual questionnaires are intended only to assess the individual tools used, such as SDS [50,51], which is used in simulation learning. However, most researchers still use questionnaires, which are compiled individually based on material reviews. Since different learning tools are used and different topics are addressed, it is not easy to choose a unique assessment instrument that could be used to assess the effectiveness of educational digital teaching tools. A similar finding in a study by Alturkistani et al. (2020) noted that, due to the diversity of topics addressed by MOOCs, it is not possible to propose a single evaluation tool for all [60].

Such differences in the use of assessment tools occur mainly because assessments must be carried out in accordance with the expected learning outcomes, which means that the whole assessment process is adapted to them [61]. The authors of various studies have also recommended different assessment methods [62]. It is also important to evaluate which material we can use to measure what we want to measure [63]. Assessments are therefore closely linked to the learning outcomes that the students expect to achieve [64].

### 4.2. Implications for Practice and Policy

Learning outcomes of students and other participants are a central part of the learning process [61]. Expected learning outcomes in students relate primarily to their knowledge, skills, and behaviors that should be achieved at the end of the educational program and measured [62].

The main positive outcomes of students’ digital behavior change support education in our study are knowledge, confidence, practical experience, and collaboration (Table 4). Active learning helps students incorporate meaningful understanding [65]. This requires students to start thinking at a higher level [66].

Increased knowledge is associated with increased self-confidence and a sense of security. Health knowledge is a key element in ensuring good quality health [67]. In a study by Albrechtsen et al. (2017) [35], the authors found that 89% of health professionals reported improved knowledge after the introduction of an intervention. In addition to increased knowledge, it is also important to improve students’ critical thinking after using digital teaching tools [47]. Students also use different approaches to improve their self-confidence in clinical skills [49].

In addition to knowledge, students also gain practical experience and preparedness for real-world situations. Additionally, simulating chronic illnesses has improved students’ perceptions of their ability to empathize and counsel persons with chronic diseases [42]. The active use of various tools has also contributed to better cooperation and interaction between students, staff, and persons with chronic diseases. Of the participants, 48% in the study by Albrechtsen et al. (2017) [35] reported increasing their professional network and collaboration during their education.

### 4.3. Restrictions on the Use of Digital Teaching Tools

Due to less familiarity with computer approaches, the challenges for the faculty are still worrying [68]. Time and material barriers were detected among the most common constraints in the analyzed studies. Bolesta et al. (2014) [37] highlighted the logistical difficulties, as, in their study, students had the option of working with only one patient simulator. It was also important to face the organization of the timing of education in the curriculum. The problem of timing in student schedules was also highlighted by other studies [43,44].

In recent years, the approach to teaching in a modified classroom has been increasingly used in undergraduate medical education [69]. The rapid development of information technology and changes in the philosophy of education has encouraged the development of the concept of a modified classroom [70]. Higher education institutions are imbued with the technological advances brought about by the industrial revolution [71], which requires fundamental changes in traditional teaching and learning activities [72]. Medical education is also changing rapidly [24]. As technology is an integral part of the work of health professionals, technology must be included in the curriculum for students [73].

Conducted systematic literature reviews have benefits for students, researchers, educators, and administrators. There are few barriers to the use of digital teaching tools. Some are related to the institution and educators’ restrictions, others to the students’ time. Institutions should provide more simulators for students to use. Additionally, students’ academic schedules should be adjusted so that students have more time for performing simulations. Therefore, students would gain more knowledge, skills, and confidence in performing simulations. Moreover, educators should undertake more education on using simulations in teaching. For educators, it is important to recognize the benefits of using digital learning tools and following trends for the sustainable development of education. Research should be focused on positive outcomes and students’ experiences with using simulations in education.

### 4.4. Limitations

A different typology of studies (qualitative, quantitative, and mixed studies) with heterogeneous results was included in this systematic review, so a meta-analysis could not be performed. There were also differences in the study’s design and the method of implementation. Different rating scales were used to assess the success of the interventions in the studies. Different populations (nursing, sports science, and pharmacy students) were included in the analyzed studies, so the results cannot be generalized for an individual population. The choice of these three target groups, which is intrinsic to the project, limited the search string; potential studies in digital behavior change support education in other areas represent an untapped resource meriting exploration in future works. In assessing the quality of the articles, despite using the MMAT rating scale, there is the possibility of subjectivity. We tried to avoid this as much as possible by involving two evaluators.

## 5. Conclusions

Using digital teaching tools such as MOOC and simulations, we can help motivate students and, thus, increase their knowledge, confidence, skills, and experience. All the studies analyzed considered only the positive effects of the use of digital learning tools that affect the effectiveness of students and their skills. Despite the topic, some limitations in the implementation of these tools in the learning process were perceived, which related mainly to their resources. The studies included in the review used a very heterogeneous set of assessment instruments. In the future, a tool should be developed to monitor the knowledge of students and health professionals to support behavior changes in persons with chronic diseases.

## Figures and Tables

**Table 1:** Inclusion and exclusion criteria.

Inclusion Criteria	
Population	Students (nursing, sports science, and pharmacy)
Intervention	RQ 1: MOOC, e-learning, simulation in the field of chronic diseases RQ 2: Assessment instruments
Outcomes	Outcomes of behavior change support education (knowledge, motivation, engagement, skills, learning outcomes, etc.)
Study design	Quantitative (e.g., case studies, randomized controlled trials, and controlled trials); qualitative (e.g., interview, questionnaire, and focus groups); and mixed method studies
Language	English language
Time frame	2000–2021
Access	/
**Exclusion criteria**	
Substantive inadequacy; records involving students from other professional fields; records in other languages; and reviews, comments, and protocols	

**Table 2:** Study characteristics and quality assessment of the included studies.

No.	Author, Year	Type of Study	MMAT Score (%)
1	Albrechtsen et al., 2017 [35]	QUAN descriptive study	80%
2	Basak et al., 2019 [36]	QUAN single-blinded RCT	90%
3	Bolesta et al., 2014 [37]	QUAN descriptive study	80%
4	Bonito 2019 [38]	QUAL study	80%
5	Bowers et al., 2017 [39]	QUAN descriptive study single-blinded, single-center, cluster RS	90%
6	Coleman & McLaughlin 2019 [40]	MMS	60%
7	Delea et al., 2010 [41]	QUAN descriptive study	70%
8	Isaacs et al., 2015 [42]	MMS	90%
9	Kolanczyk et al., 2019 [43]	MMS	80%
10	Moule et al., 2015 [44]	MMS	70%
11	Padilha et al., 2021 [45]	QUAN descriptive study	80%
12	Pharm Cowart et al., 2021 [46]	MMS	80%
13	Schultze et al., 2019 [47]	QUAN descriptive study	80%
14	Sweigart et al., 2014 [48]	MMS	50%
15	Vyas et al., 2010 [49]	QUAN descriptive study	70%

Legend: MMAT = Mixed Methods Appraisal Tool; MMS = mixed methods study; No. = number; RCT = randomized controlled trials; RS = randomized study; QUAL = qualitative; QUAN = quantitative.

**Table 3:** Instruments used for evaluating the outcomes of the research.

No.	Assessment Instruments and Short Description
1	The post-course questionnaire included nine questions. The first eight were demographic. Question 9 consisted of 15 statements that collected data on the participant’s professional benefits from the course.
2	The SSSC [50,51] includes 13 items but has been reduced to 12 due to Turkish adaptation. Participants were rated on a 5-point scale. The SDS [50,51] ordered 20 items in five subcategories. Based on the literature, a 15-item performance assessment checklist of teaching skills was prepared. The feedback form contained five questions.
3	Pre-laboratory and post-laboratory survey instrument was created using a modification of RIPLS [52] and included 19 points, which used a 5-point Likert scale to assess students’ readiness for interprofessional learning.
4	A self-administered questionnaire with open-ended questions.
5	A 15-point checklist was used to assess each appropriate insulin pen counseling and injection technique component. All elements were evaluated in the form of yes/no.
6	Short five-item anonymous pro forma consisted of four open questions and one closed question. The closed-ended questions assessed by participants on a five-point scale evaluated the learning experience. With an open-ended question, they wanted to determine students’ perceptions of what was helpful to them about this simulation, how they could improve their experience, and whether any other topic they found beneficial to include in the simulated curriculum.
7	DAS-3 [53] included 33 questions, and questions consisted of confidence in diabetes education skills had seven questions. Students answered the questions using a 5-point Likert scale
8	Data Collection Sheet Follow-Up Visit; Chronic Disease State Reflection Questions; reflections and SOAP notes. The questionnaire included 11 targeted questions on simulating chronic disease status and used a 5-point Likert scale for assessment.
9	Focus groups and surveys. The survey questionnaire included eight questions about the simulation methods used for cardiac simulations.
10	Questionnaire, review about a virtual patient, and comments.
11	The questionnaire was based on a questionnaire Davis Technology Acceptance Model [54,55] and based on ease-of-use perception [56]
12	Pre- and post-surveys questionnaire with quantitative and qualitative questions.
13	Entries data included demographic data and four specific factors necessary for determining the perception of diabetes in nursing students (number of clinical findings identified by students during the examination with the virtual patient, the total number of empathic statements shared with the virtual patient, the total number patient education statements given to the patient, and the overall outcome of the clinical inference).
14	Computerized evaluation of each of the virtual experiences.
15	Pre-simulation and post-simulation quizzes with 5–15 questions specific to each simulation scenario were used to assess whether students’ knowledge increased through participation in the simulation.

Legend: DAS-3 = Diabetes Attitude Scale; No. = number; RIPLS = Readiness for Interprofessional Learning Scale; SDS = Simulation Design Scale; SGID = Small group instructional diagnosis; SOAP = Subjective, Objective, Assessment, Plan; SSSC = Student Satisfaction and Self-Confidence in Learning Scale.

## Supplementary Materials

The following are available online at https://www.mdpi.com/article/10.3390/healthcare10010001/s1, Table S1. Search strategy in the databases. Table S2. Number of records in the databases. Table S3. List of excluded studies. Table S4. Basic data of the included studies. Table S5. PRISMA 2020 checklist. Table S6. MMAT scores.

## Abbreviations

COPD—chronic obstructive pulmonary disease; DAS-3—Diabetes Attitude Scale; MMAT—Mixed Methods Appraisal Tool; MMS—mixed method study; MOOC—Massive Open Online Course; RCS—randomized control study; RIPLS—Readiness for Interprofessional Learning Scale; RS—randomized study; SDS—Simulation Design Scale; SGID—small group instructional diagnosis; SOAP—Subjective, Objective, Assessment, Plan; SSSC—Student Satisfaction and Self-Confidence in Learning Scale; QUAL—qualitative; QUAN—quantitative.
