# Supplementary material for: Digital Tools in Behavior Change Support Education in Health and Other Students: A Systematic Review
Source: Healthcare (Basel). 2021 Dec 21;10(1):1. doi: 10.3390/healthcare10010001 (PMC8774876; doi:10.3390/healthcare10010001)
Supplement: Supplementary file 1 [file healthcare-10-00001-s001.zip › Supplementary Material 1 (Search strategy in databases).pdf]

healthcare

MDPI

Table S1. Search strategy in databases.

Database	Search string
PubMed	(("nurs*"[All Fields] AND "student*"[All Fields]) OR "health care student*"[All Fields] OR "pharmacy student*"[All Fields] OR "sport student*"[All Fields]) AND ("pedagogical method"[All Fields] OR "e learning cours*"[All Fields] OR "online cours*"[All Fields] OR "MOOC"[All Fields] OR "case stud*"[All Fields] OR "simulation*"[All Fields] OR "virtual patient*"[All Fields]) AND ("knowledge*"[All Fields] OR "motivation*"[All Fields] OR "engagement*"[All Fields] OR "skill*"[All Fields] OR "competence*"[All Fields] OR "self-care"[All Fields] OR "self-management"[All Fields] OR (("change"[All Fields] OR "changed"[All Fields] OR "changes"[All Fields] OR "changing"[All Fields] OR "changings"[All Fields]) AND ("behavior"[MeSH Terms] OR "behavior"[All Fields] OR "behavioral"[All Fields] OR "behavioural"[All Fields] OR "behavior s"[All Fields] OR "behaviorally"[All Fields] OR "behaviour"[All Fields] OR "behaviourally"[All Fields] OR "behaviours"[All Fields] OR "behaviors"[All Fields] OR "pattern"[All Fields] OR "pattern s"[All Fields] OR "patternability"[All Fields] OR "patternable"[All Fields] OR "patterned"[All Fields] OR "patterning"[All Fields] OR "patternings"[All Fields] OR "patterns"[All Fields])) OR "change attitudes"[All Fields] OR "behaviour change"[All Fields] OR "behavior change"[All Fields] OR "behaviour change techniques"[All Fields] OR "behavior change techniques"[All Fields] OR "health behaviour"[All Fields] OR "health behavior"[All Fields]) AND ("non-communicable disease"[All Fields] OR "chronic disease*"[All Fields] OR "chronic illness"[All Fields] OR "coronary disease"[All Fields] OR "coronary artery disease"[All Fields] OR "heart disease"[All Fields] OR "heart failure"[All Fields] OR "cardiovascular disease"[All Fields] OR "high blood pressure"[All Fields] OR "hypertension"[All Fields] OR "diabetes mellitus type 2"[All Fields] OR "ischemic heart disease"[All Fields] OR "type 2 diabetes"[All Fields] OR "non-insulin-dependent diabetes"[All Fields] OR "adult-onset diabetes"[All Fields] OR "NIDDM"[All Fields] OR "T2D"[All Fields] OR "obesity"[All Fields])
CINAHL	"nurs* student*" OR "health care student*" OR "pharmacy student*" OR "sport student*" AND "pedagogical method" OR "e-learning cours*" OR "online cours*" OR "MOOC" OR "case stud*" OR "simulation*" OR "virtual patient*" AND "knowledge*" OR "motivation*" OR "engagement*" OR "skill*" OR "competence*" OR "self-care" OR "self-management" OR "change the behaviour" OR "change attitudes" OR "behaviour change" OR "behavior change" OR "behaviour change techniques" OR "behavior change techniques" OR "health behaviour" OR "health behavior" AND "non-communicable disease" OR "chronic disease*" OR "chronic illness" OR "coronary disease" OR "coronary artery disease" OR "heart disease" OR "heart failure" OR "cardiovascular disease" OR "high blood pressure" OR "hypertension" OR "diabetes mellitus type 2" OR "ischemic heart disease" OR "type 2 diabetes" OR "non-insulin-dependent diabetes" OR "adult-onset diabetes" OR "NIDDM" OR "T2D" OR "obesity"
MEDLINE	"nurs* student*" OR "health care student*" OR "pharmacy student*" OR "sport student*" AND "pedagogical method" OR "e-learning cours*" OR "online cours*" OR "MOOC" OR "case stud*" OR "simulation*" OR "virtual patient*" AND "knowledge*" OR "motivation*" OR "engagement*" OR "skill*" OR "competence*" OR "self-care" OR "self-management" OR "change the behaviour" OR "change attitudes" OR "behaviour change" OR "behavior change" OR "behaviour change techniques" OR "behavior change techniques" OR "health behaviour" OR "health behavior" AND "non-communicable disease" OR "chronic disease*" OR "chronic illness" OR "coronary disease" OR "coronary artery disease" OR "heart disease" OR "heart failure" OR "cardiovascular disease" OR "high blood pressure" OR "hypertension" OR "diabetes mellitus type 2" OR "ischemic heart disease" OR "type 2 diabetes" OR "non-insulin-dependent diabetes" OR "adult-onset diabetes" OR "NIDDM" OR "T2D" OR "obesity"
Web of Science	((("nurs* student*" OR "health care student*" OR "pharmacy student*" OR "sport student*") AND "pedagogical method" OR "e-learning cours*" OR "online cours*" OR "MOOC" OR "case stud*" OR "simulation*" OR "virtual patient*") AND "knowledge*" OR "motivation*" OR "engagement*" OR

	"skill*" OR "competence*" OR "self-care" OR "self-management" OR "change the behaviour" OR "change attitudes" OR "behaviour change" OR "behavior change" OR "behaviour change techniques" OR "behavior change techniques" OR "health behaviour" OR "health behavior") AND "non-communicable disease" OR "chronic disease*" OR "chronic illness" OR "coronary disease" OR "coronary artery disease" OR "heart disease" OR "heart failure" OR "cardiovascular disease" OR "high blood pressure" OR "hypertension" OR "diabetes mellitus type 2" OR "ischemic heart disease" OR "type 2 diabetes" OR "non-insulin-dependent diabetes" OR "adult-onset diabetes" OR "NIDDM" OR "T2D" OR "obesity"
Scopus	(TITLE-ABS-KEY (("nurs* student*" OR "health care student*" OR "pharmacy student*" OR "sport student*")) AND TITLE-ABS-KEY (("pedagogical method" OR "e-learning cours*" OR "online cours*" OR "MOOC" OR "case stud*" OR "simulation*" OR "virtual patient*")) AND TITLE-ABS-KEY (("knowledge*" OR "motivation*" OR "engagement*" OR "skill*" OR "competence*" OR "self-care" OR "self-management" OR "change the behaviour" OR "change attitudes" OR "behaviour change" OR "behavior change" OR "behaviour change techniques" OR "behavior change techniques" OR "health behaviour" OR "health behavior")) AND TITLE-ABS-KEY (("non-communicable disease" OR "chronic disease*" OR "chronic illness" OR "coronary disease" OR "coronary artery disease" OR "heart disease" OR "heart failure" OR "cardiovascular disease" OR "high blood pressure" OR "hypertension" OR "diabetes mellitus type 2" OR "ischemic heart disease" OR "type 2 diabetes" OR "non-insulin-dependent diabetes" OR "adult-onset diabetes" OR "NIDDM" OR "T2D" OR "obesity")))
SAGE	[[All "nurs* student*"] OR [All "health care student*"] OR [All "pharmacy student*"] OR [All "sport student*"]] AND [[All "pedagogical method"] OR [All "e-learning cours*"] OR [All "online cours*"] OR [All "mooc"] OR [All "case stud*"] OR [All "simulation*"] OR [All "virtual patient*"]] AND [[All "knowledge*"] OR [All "motivation*"] OR [All "engagement*"] OR [All "skill*"] OR [All "competence*"] OR [All "self-care"] OR [All "self-management"] OR [All "change the behaviour"] OR [All "change attitudes"] OR [All "behaviour change"] OR [All "behavior change"] OR [All "behaviour change techniques"] OR [All "behavior change techniques"] OR [All "health behaviour"] OR [All "health behavior"]] AND [[All "non-communicable disease"] OR [All "chronic disease*"] OR [All "chronic illness"] OR [All "coronary disease"] OR [All "coronary artery disease"] OR [All "heart disease"] OR [All "heart failure"] OR [All "cardiovascular disease"] OR [All "high blood pressure"] OR [All "hypertension"] OR [All "diabetes mellitus type 2"] OR [All "ischemic heart disease"] OR [All "type 2 diabetes"] OR [All "non-insulin-dependent diabetes"] OR [All "adult-onset diabetes"] OR [All "niddm"] OR [All "t2d"] OR [All "obesity"]]
Cochrane Library	"nurs* student*" OR "health care student*" OR "pharmacy student*" OR "sport student*" AND "pedagogical method" OR "e-learning cours*" OR "online cours*" OR "MOOC" OR "case stud*" OR "simulation*" OR "virtual patient*" AND "knowledge*" OR "motivation*" OR "engagement*" OR "skill*" OR "competence*" OR "self-care" OR "self-management" OR "change the behaviour" OR "change attitudes" OR "behaviour change" OR "behavior change" OR "behaviour change techniques" OR "behavior change techniques" OR "health behaviour" OR "health behavior" AND "non-communicable disease" OR "chronic disease*" OR "chronic illness" OR "coronary disease" OR "coronary artery disease" OR "heart disease" OR "heart failure" OR "cardiovascular disease" OR "high blood pressure" OR "hypertension" OR "diabetes mellitus type 2" OR "ischemic heart disease" OR "type 2 diabetes" OR "non-insulin-dependent diabetes" OR "adult-onset diabetes" OR "NIDDM" OR "T2D" OR "obesity"
