# Supplementary material for: Digital Tools in Behavior Change Support Education in Health and Other Students: A Systematic Review
Source: Healthcare (Basel). 2021 Dec 21;10(1):1. doi: 10.3390/healthcare10010001 (PMC8774876; doi:10.3390/healthcare10010001)
Supplement: Supplementary file 1 [file healthcare-10-00001-s001.zip › Supplementary Material 2 (Number of records in databases).pdf]

healthcare MDPI

Supplementary Material 2

Table S2. Number of records in databases.

#	Keywords	PubMed	CINAHL	MEDLINE	Web of Science	SAGE	Scopus	Cochrane Library
1	"nurs* student*" OR "health care student*" OR "pharmacy student*" OR "sport student*"	68.339	25.860	22.786	21.332	69	40.470	190
2	"pedagogical method" OR "e-learning cours*" OR "online cours*" OR "MOOC" OR "case stud*" OR "simulation*" OR "virtual patient*"	664.826	133.870	662.744	3.576.738	128.005	4.962.420	12.310
3	"knowledge*" OR "motivation*" OR "engagement*" OR "skill*" OR "competence*" OR "self-care" OR "self-management" OR "change the behaviour" OR "change attitudes" OR "behaviour change" OR "behavior change" OR "behaviour change techniques" OR "behavior change techniques" OR "health behaviour" OR "health behavior"	2.106.179	641.402	1.451.603	2.896.042	1.097.901	3.701.711	99.979
4	"non-communicable disease" OR "chronic disease*" OR "chronic illness" OR "coronary disease" OR "coronary artery disease" OR "heart disease" OR "heart failure" OR "cardiovascular disease" OR "high blood pressure" OR "hypertension" OR "diabetes mellitus type 2" OR "ischemic heart disease" OR "type 2 diabetes" OR "non-insulin-dependent diabetes" OR "adult-onset diabetes" OR "NIDDM" OR "T2D" OR "obesity"	1.892.886	561.852	2.019.238	1.758.741	211.808	2.670.902	225.030
	#1 AND #2 AND #3 AND #4	66	22	34	37	15	62	0
