# Supplementary material for: Digital Tools in Behavior Change Support Education in Health and Other Students: A Systematic Review
Source: Healthcare (Basel). 2021 Dec 21;10(1):1. doi: 10.3390/healthcare10010001 (PMC8774876; doi:10.3390/healthcare10010001)
Supplement: Supplementary file 1 [file healthcare-10-00001-s001.zip › Supplementary Material 3 (List of excluded studies).pdf]

healthcare

MDPI

Supplementary Material 3

Table S3. List of excluded studies.

Author, year	Exclusion reason
Wu & Lin, 2011	Language
Holzemer, et al., 1986	Year of publication
Cain & Piascik, 2015	Inappropriate type of study
Newton & Krebs, 2020	Inappropriate type of study
Vaona, et al., 2018	Inappropriate type of study
Hernandez, 2019	Inappropriate type of study
Harris, et al., 2020	Inappropriate type of study
Miller, et al., 2020	Inappropriate type of study
Lawn, et al., 2017	Inappropriate type of study
Dorri, et al., 2019	Substantive inadequacy
Tawalbeh, 2020	Substantive inadequacy
van de Mortel, et al., 2017	Substantive inadequacy
Yousey & Faulkner, 2010	Substantive inadequacy
Romero-Collado, et al., 2020	Substantive inadequacy
Luo & Kalman, 2018	Substantive inadequacy
Dicks & Romanelli, 2019	Substantive inadequacy
Luo, et al., 2019	Substantive inadequacy
Consorti, et al., 2012	Substantive inadequacy
Isaza-Restrepo, et al., 2018	Substantive inadequacy
Kononowicz, et al., 2019	Substantive inadequacy
Barnett, et al., 2016	Substantive inadequacy
Walker & Gantt, 2010	Substantive inadequacy
Padilha, et al., 2019	Substantive inadequacy
Zeitoun, et al., 2014	Substantive inadequacy
Dukhi, et al., 2014	Substantive inadequacy
Odajima & Furuichi, 2020	Substantive inadequacy
Sandstrom, 2006	Not available in full text
Dacey, et al., 2010	Not available in full text
Scherer, et al., 2007	Not available in full text
Tilton, et al., 2015	Not available in full text
Mauro, et al., 2018	Not available in full text
