# Supplementary material for: Digital Tools in Behavior Change Support Education in Health and Other Students: A Systematic Review
Source: Healthcare (Basel). 2021 Dec 21;10(1):1. doi: 10.3390/healthcare10010001 (PMC8774876; doi:10.3390/healthcare10010001)
Supplement: Supplementary file 1 [file healthcare-10-00001-s001.zip › Supplementary Material 4 (Basic characteristics of the included studies).pdf]

healthcare

MDPI

Supplementary Material 4

Table S4. Basic characteristics of the included studies.

No.	Study sample	Aim	Findings	Digital digital teaching tools	Subject of digital teaching tools
**1**	52.006 participants registered for one of the three MOOC courses, of which 29.469 participants actively participated. 1.303 participants completed the questionnaire. Of these, 845 were health professionals (34% physicians, 15% researchers, 11% nurses, and 8% students).	The study aimed to compare the impact of participation in an online course on the prevention and treatment of diabetes and obesity on the knowledge, skills, and careers of health professionals from a developing country compared to developed countries.	- More than 80% report on educational benefits, improved knowledge, working life, and practice.<br>- More than 40% of them reported that their professional network had expanded.<br>- 48% of health professionals reported increasing their network of professionals.<br>- 36% of the course participants collaborated with the rest through discussion forums and Facebook.<br>- 89% of students reported the benefits of the course in terms of career and professional performance.<br>- 89% of health professionals reported acquiring knowledge related to their future careers.<br>- Participants from developing countries had a more significant impact on their clinical practice than health professionals from developed countries.	- The MOOC Diabetes - a Global Challenge.<br>- MOOC contains reading materials, video lectures, and tests. Based on the tests and tasks, the participants determine the achieved level.	Diabetes (epidemiology, prevention, obesity, pharmacological treatment, genetic forms, etc.).

**2**	The survey was conducted with 132 second-year nursing school students. The criteria for inclusion in the study were met by 71 students (36 control groups and 35 intervention groups).	Compare the effectiveness of simulation and theoretical lecture in terms of improving patient teaching skills of nursing students.	- The overall assessment of learning abilities was statistically significant among the control group students and among the students who used the simulation ($p \leq 0.01$).<br>- The difference between students' self-confidence while teaching patients to use inhalers was statistically significant ($p \leq 0.01$).	- Standard patients.<br>- Two standardized patients with similar socio-demographic characteristics and medical history were used. A meeting with standardized patients took place three days before the simulation.	Inhaler drug use.
**3**	Seventy-five students agreed to participate (61 in the field of pharmacy, 13 in nursing, and 1 participant who did not specify the area). Seventy-two participated in the pre-laboratory questionnaire, of which 65 also participated in the post-laboratory test (56 in the field of pharmacy and 9 in nursing).	The study aimed to describe the design, implementation, and results of interprofessional education. The aim was for students to identify the circumstances in which interprofessional care would improve patient outcomes and develop effective collaboration.	The curricular intervention's effects on students' knowledge and skills were not significant in comparing the results.<br>- The difference in scores became significant for all six criteria using the retrospective pre-test / post-test methodology.<br>- No significant improvement was observed in all survey items.<br>- Students gained the greatest experience in communication skills.<br>- Overall results have been improved.	- Human patient simulation.<br>- Before the simulation, students were given instructions to read the literature. They also had access to the necessary patient data.	Clinical laboratory with a scenario of acute exacerbation of heart failure. Students were involved in diagnosis and treatment.
**4**	106 nursing with no previous experience in the virtual study.	Describe the usefulness of multimedia case study software to facilitate student learning in a clinical practicum.	- Students commented that the software helped guide students in performing tasks in an actual clinical practicum.<br>- Some also commented on the usefulness of the resources.<br>- The software helped to encourage critical thinking. They also claim that the software has been beneficial in preparing for the clinical practicum.	- Virtual Case Study.	Chronic heart failure, stroke, breast cancer, COPD, dementia.

**5**	103 pharmacy students (48 control group, and 55 intervention group). Two students were absent and dropped out of the survey as a result.	Compare results before and after procedure and counseling skills among students with or without simulated patients.	- The intervention group showed an increase from baseline results of tests (p = 0.077) compared to the control group.<br>- The intervention team received a significantly higher rating in the field of advice.<br>- The overall assessment of knowledge retention was higher for the intervention team but was not statistically significant.	- Standardized simulated patients.	Insulin injection technique and counseling skills.
**6**	20 nursing students.	Evaluate the simulated role-playing scenario provided to nursing students to support teacher skills development.	- Students rated their learning experiences on a five-point scale. Eighteen students rated their experience as excellent, and two students as very good.	- Simulated patients.	COPD.
**7**	187 third-year pharmacy students were included in the research. 110 students attended traditional lectures and laboratory exercises without participating in the simulation, so they were classified into a control group. 77 students were included in the experimental group. Of these, 57 performed more than half of the instruments.	Assess students' attitudes and confidence about education about diabetes before and after participating in the simulation.	- The most significant increase has been found in the area of trust in using the glucometer and advising patients on diabetes medicines.<br>- A higher percentage of students in the experimental group trusted all aspects of the assessed skills on diabetes education.<br>- The most significant difference in trust occurred in making recommendations to other hhealthcare providers on diabetes management and the general ability to provide education to people with diabetes.	- Simulation.	Diabetes.

**8**	130 pharmacy students (120 students completed the survey).	Assess the impact of the simulation on students ʼperceptions of their empathy and their counseling skills.	- On average, the reflection scores were 98.5%.<br>- The rates of reflection were similar from year to year, although, in 2014, we found a significant difference compared to the cohorts of 2011, 2012, and 2013.	- Simulation.	Ambulatory care.
**9**	12 pharmacy students.	Describe how simulation learning is applied to cardiac topics in pharmaceutical schools and further discuss the challenges and successes of these simulations.	- The topics highlighted by the authors in the context of qualitative analysis were: common; simulation formats; interprofessional education; challenges; strategies for success; and the benefits of simulation learning.<br>- The most common obstacles to the simulation included time in students' schedules, financial resources, space, and faculty development time activities.	- Simulation.	Cardiac life support, heart failure, hypertension, and transitions of care.
**10**	12 participant (five qualified nurses, four students, three others) and 20 (13 qualified nurses, Five students, two other).	Develop online simulation scenarios to meet the learning needs of nurses and health professionals.	- 69.1% of individuals reported previous experience with online learning.<br>- 87.0% of respondents that they could benefit from online learning.<br>- Participants in both scenarios identified the positive aspects of both scenarios: comfort, ease of use, quality of content, interactivity, and impact on practice.	- Virtual patients.	Prostate cancer.
**11**	93 participants of which 61 were nursing students, 14 were nurses and 18 were specialist rehabilitation nurses	The aim was to assess the usefulness and readiness of nursing students and nurses for MOOC, which aimed to improve the self-management skills of COPD patients.	- Participants considered the MOOC to be beneficial for education and lifelong learning.<br>- Older nurses scored higher than younger nurses and students (χ^2 (2, n = 93) = 8,43, p = 0,015, ε^2 = 0,092).	- MOOC.	COPD.

**12**	87 students of Doctor of Pharmacy.	Assess the confidence of 1st year students in their skills and techniques through simulation.	- Improvements in confidence in the implementation of activities were present in students [4.13 ± 0.70 vs. 2.75 ± 0.99].<br>- Most of the students who participated in the simulation agreed or strongly agreed that the simulation represents valuable.<br>- Students perceived problems using the technology.	- Simulation.	Hypertension/drug information.
**13**	8.473 entries from nursing students from 116 institutions across 38 states.	Use basic data mining techniques and find out how disease awareness programs work on knowledge.	- Age and experience influence the way the patient is examined.<br>- Older and more experienced nursing students report fewer clinical findings and generally have a lower simulation score.<br>- Older students conduct a less thorough interview.<br>- Older and more experienced nursing students make more empathetic and instructive statements to the virtual patient.	- Virtual patient Software Shadow Health Digital Clinical Experience™.	Diabetes.
**14**	800 nursing students.	Evaluate the effectiveness of interviewing with avatars in a virtual simulation.	- Students who interviewed with a patient with an avatar in a virtual environment asked an average of 10.06 questions ranging from 0 to 24.<br>- Student self-evaluation supported the effectiveness of this different experience.	- Virtual Simulations.	Mental health.
**15**	28 pharmacy students; 27 completing all materials.	Introduce a five-year curriculum simulation by a doctor of pharmacy to demonstrate a hybrid model for conducting introductory experience with the pharmaceutical practice.	- Students' grades on the quiz based on knowledge were significantly higher than those on the quiz before the simulation.<br>- Knowledge retention was significantly higher among simulation participants ($p = 0.004$).<br>- The majority of students (76%) felt more confident after the simulation series.<br>- 96% of participants agreed that simulations should be offered every year.	- Simulation.<br>- Simulation scenarios included preparations for, for example, a clinical meeting and reporting.	Asthma exacerbation, acute decompensated heart failure, and infective endocarditis with a subsequent anaphylactic reaction to the antibiotic.

healthcare MDPI

	- 93%, however, considered simulation training to be enhanced clinical learning compared to standard lectures.	

Legend: COPD = chronic obstructive pulmonary disease; MOOC = Massive Open Online Course
