# Supplementary material for: Digital Tools in Behavior Change Support Education in Health and Other Students: A Systematic Review
Source: Healthcare (Basel). 2021 Dec 21;10(1):1. doi: 10.3390/healthcare10010001 (PMC8774876; doi:10.3390/healthcare10010001)
Supplement: Supplementary file 1 [file healthcare-10-00001-s001.zip › Supplementary Material 6 (MMAT Score).pdf]

healthcare

MDPI

Supplementary Material 6

Table S6. MMAT Score

		QUAL study					QUAN randomaized study					QUAN descriptive study					MMS				
**No.**	**Author, year**	**1.1.**	**1.2.**	**1.3.**	**1.4.**	**1.5.**	**2.1.**	**2.2.**	**2.3.**	**2.4.**	**2.5.**	**4.1.**	**4.2.**	**4.3.**	**4.4.**	**4.5.**	**5.1.**	**5.2.**	**5.3.**	**5.4.**	**5.5.**
**1**	Albrechtsen, et al., 2017 [35]											1	1	1	0	1					
**2**	Basak, et al., 2019 [36]						1	1	1	0.5	1										
**3**	Bolesta, et al., 2014 [37]											1	1	1	0	1					
**4**	Bonito, 2019 [38]	1	1	1	1	0															
**5**	Bowers, et al., 2017 [39]						0.5	1	1	1	1										
**6**	Coleman & McLaughlin, 2019 [40]	1	1	1	0	1						0.5	0.5	1	0.5	1	1	1	0.5	0.5	0
**7**	Delea, et al., 2010 [41]											1	0.5	1	0	1					
**8**	Isaacs, et al., 2015 [42]	1	1	1	1	0.5						1	1	1	1	0.5	1	1	1	1	0.5
**9**	Kolanczyk, et al., 2019 [43]	1	0.5	0.5	1	0.5						1	0.5	0.5	1	1	1	1	1	0.5	0.5
**10**	Moule, et al., 2015 [44]	1	1	1	0.5	0						1	1	1	0.5	0	1	1	1	0.5	0
**11**	Padilha, et al., 2021 [45]											1	1	1	0	1					
**12**	Pharm Cowart, et al., 2021 [46]	1	1	1	1	0						1	1	1	1	0	1	1	1	1	0
**13**	Schultze, et al., 2019 [47]											1	1	1	0	1					
**14**	Sweigart, et al., 2014 [48]	1	1	0.5	0.5	1						1	1	0.5	0.5	1	1	1	0.5	0	0
**15**	Vyas, et al., 2010 [49]											0.5	1	1	0	1					
